# Microfluidic One-Pot Digital Droplet FISH Using LNA/DNA Molecular Beacons for Bacteria Detection and Absolute Quantification

**DOI:** 10.3390/bios12040237

**Published:** 2022-04-12

**Authors:** Yu-Ting Kao, Silvia Calabrese, Nadine Borst, Michael Lehnert, Yu-Kai Lai, Franziska Schlenker, Peter Juelg, Roland Zengerle, Piotr Garstecki, Felix von Stetten

**Affiliations:** 1 Laboratory for MEMS Applications, IMTEK-Department of Microsystems Engineering, University of Freiburg, Georges-Koehler-Allee 103, 79110 Freiburg, Germany; yu-ting.kao@imtek.uni-freiburg.de (Y.-T.K.); nadine.borst@hahn-schickard.de (N.B.); yu-kai.lai@imtek.uni-freiburg.de (Y.-K.L.); roland.zengerle@imtek.uni-freiburg.de (R.Z.); 2 Institute of Physical Chemistry, Polish Academy of Sciences, Kasprzaka 44/52, 01-224 Warsaw, Poland; garst@ichf.edu.pl; 3 Hahn-Schickard, Georges-Koehler-Allee 103, 79110 Freiburg, Germany; silvia.calabrese@hahn-schickard.de (S.C.); michael.lehnert@hahn-schickard.de (M.L.); franziska.schlenker@hahn-schickard.de (F.S.); peter.juelg@hahn-schickard.de (P.J.)

**Keywords:** fluorescence *in situ* hybridization, locked nucleic acid, molecular beacons, droplet microfluidics, amplification-free detection, digital assays

## Abstract

We demonstrate detection and quantification of bacterial load with a novel microfluidic one-pot wash-free fluorescence *in situ* hybridization (FISH) assay in droplets. The method offers minimal manual workload by only requiring mixing of the sample with reagents and loading it into a microfluidic cartridge. By centrifugal microfluidic step emulsification, our method partitioned the sample into 210 pL (73 µm in diameter) droplets for bacterial encapsulation followed by *in situ* permeabilization, hybridization, and signal detection. Employing locked nucleic acid (LNA)/DNA molecular beacons (LNA/DNA MBs) and NaCl-urea based hybridization buffer, the assay was characterized with *Escherichia coli*, *Klebsiella pneumonia*, and *Proteus mirabilis*. The assay performed with single-cell sensitivity, a 4-log dynamic range from a lower limit of quantification (LLOQ) at ~3 × 10^3^ bacteria/mL to an upper limit of quantification (ULOQ) at ~3 × 10^7^ bacteria/mL, anda linearity R^2^ = 0.976. The total time-to-results for detection and quantification was around 1.5 hours.

## 1. Introduction

Detection and enumeration of bacteria are important in various fields, such as medicine [1], public health [2], food safety [3], and water quality control [4]. The presence of bacteria and their concentration must be determined in order to assess and control health hazards to humans, prevent bacterial outbreaks, diagnose bacterial infections, provide a proper treatment and further avoid antibiotic abuse. Current widely used culture-based gold standards for the detection and enumeration of bacteria relies on manually making dilution series of the sample and cultivating them on agar plates, followed by counting the colony forming units (CFU) [5]. However, this standard method is time-consuming and labor intensive. Although laboratory automation, such as automated spiral plating [6] increases lab throughput by omitting manual dilutions and plating steps, bacteria counting can only be performed after extended incubation, e.g., 18 to 24 hours, to enable colonies of sufficient size for optical counting. These culture-based methods only count the bacteria that grow on the agar plates and underestimate viable but non-culturable (VBNC) bacteria [7] that pose a risk to public health [8,9].

In contrast to culture-based methods, rapid molecular methods can detect both culturable and non-culturable bacteria without an extended incubation time. Among the molecular methods, quantitative real-time polymerase chain reaction (real-time PCR or qPCR) has been demonstrated to be much less time-consuming for identifying and enumerating microbes from complex backgrounds [10,11]. However, DNA extraction is a crucial step in qPCR [12,13]. The presence of inhibitors in complex samples and in the extracted DNA may lead to unreliable results [14,15], especially in samples with low concentrations of the analytes. In addition, the relative quantification of bacteria requires the generation of additional standard curves [10,11,16,17] to obtain the number of bacteria. This number of bacteria is usually obtained from conventional counting methods, which are prone to errors because of the dilution series.

Fluorescence *in situ* hybridization (FISH) is a molecular cytogenetic technique based on the selective binding of fluorescence-labelled oligo-nucleotide probes to complementary DNA or RNA sequences inside the cells. It is also widely used for microbial identification by targeting ribosomal RNA (rRNA) [18,19]. As an advantage, this technique does not require nucleic acid isolation and is amplification-free without the risks of false-positive results caused by amplicon carry-over, which may occur in amplification based methods such as PCR [14]. On the other hand, a FISH protocol still requires a number of processing steps for fixation/permeabilization, hybridization, and several washing steps to remove excess unbound probes [20]. Insufficient washing can result in high background fluorescence signals, which makes target detection difficult. The enumeration of bacteria by FISH is commonly based on fluorescence microscopy or flow cytometry [19]. Although microscopic counting is frequently used, reliable statistical analyses require that a minimum of 20 random microscopic fields, or a minimum of 350 bacteria, are counted on a glass slide [21]. Even with the help of a counting chamber, particularly a hemocytometer, there is a lower limit for an accurate count of 2.5 × 10^5^ bacteria/mL [22]. Flow cytometers serve as high throughput counting tools that count at least 1000 particles per second [23]. Nevertheless, flow cytometers are often expensive, bulky, and do not provide a visual check for the counted bacteria. In addition, for a direct bacterial count, precise control of the flow and recording of the sample volume is necessary, which is not a feature of all flow cytometers.Accordingly, additional reference beads are required for absolute bacterial counting [24]. To this end, there is a need to further improve the FISH technique and facilitate its quantification method.

Droplet digital assays provide an opportunity for absolute quantification of bacteria with reduced time-to-results compared to plate counting methods [25,26,27,28,29]. Bacteria samples are partitioned into droplets, which results in a statistical distribution. By counting the portion of droplets containing bacteria among the total number of droplets, the bacterial concentration can be determined by Poisson distribution [30]. Most droplet digital assays for bacterial quantification measured the growth of bacteria through either metabolism [25,26,29] or turbidity [27], whereas direct droplet digital PCR (dddPCR) [28] tookadvantage of PCR but omitted the DNA extraction steps by lysing the bacteria directly in the droplets. However, performing PCR requires thermal cycling equipment. In contrast, isothermal amplification, such as loop-mediated isothermal amplification (LAMP), is not suitable for high degrees of multiplexing [31]. Both amplification methods are challenging when designing a multiplex assay since the primer pairs and probes have to be adapted to other primer pairs to decrease mutual complementarity [20]. In contrast, FISH only requires the design of one single probe per target.

In order to perform a FISH assay in droplets, molecular beacons (MBs) offer the potential of avoiding the washing steps since the fluorescent signal is only emitted upon binding to the target sequences [32]. This mechanism allows MBs to detect nucleic acid targets without the separation of excess probes/reagents in a FISH assay. Researchers have developed an amplification-free assay by using peptide nucleic acid (PNA) MBs binding to a bacterial lysate in droplets [33,34]. PNA is a synthetic DNA analogue that has a neutral peptide backbone instead of the deoxyribose phosphate backbone of DNA [35]. The non-charged nature of the PNA backbone improves thermal stability, has more rapid hybridization kinetics, and has higher specificity compared with DNA probes [36]. However, PNA has been shown to have a propensity to self-aggregate and fold that interferes with duplex formation [37]. Moreover, its solubility varies with the PNA sequence [38]. Another possibility for improving the thermal stability of hybridization is using locked nucleic acid (LNA) [39]. LNA is an RNA analogue that has higher solubility rates than PNA. Therefore, LNA MBs could be a good candidate for the integration of the FISH assay in droplets for absolute quantification of bacteria. Even though the hybridization kinetics of the LNA probes are relatively slow when incorporated into MB [40], the high energy barrier for opening the MB can be overcome by adjusting the stem length, the number of integrated LNA monomers, and the sequence composition [37].

**Figure 1:**
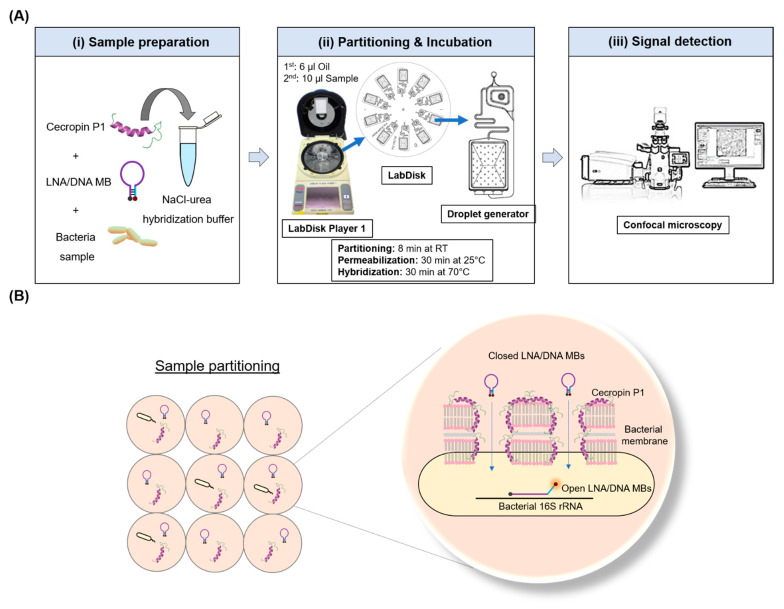
Microfluidic one-pot digital droplet-FISH assay. (**A**) Detailed workflow of the microfluidic one-pot digital droplet-FISH assay. (i) Sample preparation: cecropin P1, LNA/DNA MBs, and bacteria were mixed in hybridization buffer. (ii) The sample mixture was then added into the LabDisk for partitioning after the first injection and centrifugation of oil. The LabDisk Player 1 was programmed for permeabilization at 25 °C for 30 min and for hybridization at 70 °C for 30 min. (iii) Fluorescence and bright field images were taken directly from the chamber of the LabDisk by confocal microscopy for droplet analysis. (**B**) Mechanism of the one-pot FISH assay in a partitioned sample. The sample mixture was partitioned into ~47,600 droplets. In a single droplet, closed LNA/DNA MBs entered the bacteria through the pores made by cecropin P1 and then open their stems during binding to bacterial 16S rRNA, resulting in a fluorescence signal.

In this paper, we demonstrate a workflow for a wash-free digital droplet-FISH assay, merging the advantages of a simple probe design, multiplexing potential, minimum preparatory steps, a short incubation time, and the absolute quantification of bacteria. To our knowledge, there are no quantitative and fully integrated FISH assays taking advantage of the power of droplet microfluidics at present. The novel one-pot digital droplet-FISH assay is presented in Figure 1, which comprises partitioning the bacteria into droplets by centrifugal microfluidic step emulsification, *in situ* cell permeabilization, probe-to-target hybridization, and fluorescence signal detection. Our guidelines for the design and optimization of the high affinity LNA/DNA molecular beacons is provided in the Supplementary Materials.

## 2. Materials and Methods

### 2.1. Bacteria Cultivation

*Escherichia coli* (*E. coli*, DSM 6897), *Klebsiella pneumonia* (*K. pneumonia*, DSM 30104), and *Proteus mirabilis* (*P. mirabilis*, DSM 4479) were cultured in 1.5% Luria-Bertani (LB) (Carl Roth, Germany) agar plates overnight at 37 °C. Cultures were started from single colonies in LB broth. These overnight cultured bacterial suspensions were subcultured in fresh LB broth to mid-log phase for experiments.

### 2.2. LNA/DNA MB Design

The sequences of the designed MBs were modified from probe EUB338 [18]. Nucleotides, complementary to the 3′ end of the probe, were added to the 5′ end of the original probe, so that a stem-loop structure could form. Furthermore, LNA monomers were incorporated every third base into the MBs since it has been shown to refrain from RNA degradation and exhibit faster hybridization kinetics with greater signal enhancement [37]. The LNA incorporating DNA MBs are referred as LNA/DNA MBs here. The optimal LNA/DNA MB sequence used in this study was ATTO647N-TCC**GCTGCCTCCCGTAGGA**-BHQ-2. Nucleotides in bold target the domain *Bacteria* (S-D-Bact-0338-a-A-18 [18]); letters in red are LNA monomers; letters in black are DNA monomers; and the underlined nucleotides form a stem structure due to self-complementarity. The LNA/DNA MBs were synthesized by Integrated DNA Technologies (Coralville, IA, USA).

### 2.3. Microfluidic One-Pot Digital Droplet-FISH Workflow on a LabDisk

The workflow for microfluidic one-pot digital droplet-FISH (Figure 1A) is as follows: 1 µL of the sample (bacterial culture) is mixed with 9 µL of hybridization and permeabilization buffer containing 0.02 mM Tris-HCl (pH 7.0) (Thermo Fisher Scientific, Darmstadt, Germany), 900 mM NaCl (Promega, Walldorf, Germany), 1 M urea (Thermo Fisher Scientific, Germany), 60 µM cecropin P1 (CP1) (Bachem, Bubendorf, Switzerland), 25 nM LNA/DNA MBs (Integrated DNA Technologies, Coralville, IA, USA) in a 0.2 mL PCR tube (QIAGEN, Hilden, Germany) by pipetting. The microfluidic cartridge (LabDisk) used for partitioning was actuated by a customized centrifuge equipped with temperature control (LabDisk Player 1, Figure S1A, DIALUNOX, formerly QIAGEN Lake Constance, Germany). Details and configurations of the LabDisk are shown in Figure S1B. Partitioning of the sample into ~47,600 droplets with a diameter of 73 µm (210 pL) was performed at room temperature (RT) by centrifugal step emulsification as described in previous work [41]. The size and volume distribution of the droplets are shown in Figure S1C,D. To prepare the LabDisk for partitioning of the sample, 6 μL of 3M^TM^ Novec^TM^ 7500 fluorinated oil containing 5% dSURF surfactants (Fluigent, Le Kremlin-Bicêtre, France) were pre-loaded into each inlet of the droplet generation units and centrifuged at 40 Hz (acceleration 15 Hz/s) for 2 minutes. Next, 10 μL of each sample mixed with hybridization and permeabilization buffer were loaded and centrifuged at 18.5 Hz (acceleration 40 Hz/s) for 8 minutes for droplet generation. The cartridge was incubated for 30 min at 25 °C for permeabilization of bacteria by CP1 and then a further 30 minutes at 70 °C for hybridization of the MBs with bacterial 16S rRNA.

### 2.4. Detection of Single Bacteria in Droplets and Data Processing for Absolute Quantification

Fluorescence images at excitation/emission wavelengths of 633/685 nm and bright field images were taken by confocal microscopy (LSM 880 Observer, ZEISS, Oberkochen, Germany). Bright field images were used for the analysis of droplet numbers and size, and were analyzed by a customized MATLAB [42] (The MathWorks, Natick, MA, USA) program (Figure S2). Fiji [43] with the function of “Cell Counter” was used to count (mark) the droplets that contained bacteria (red fluorescence dots) (Figure S3), which were considered positive droplets.

The concentration of bacteria was calculated by Poisson distribution. Around 4,000 to 5,000 droplets were analyzed in each reaction chamber. The total number of analyzed droplets (N) and the number of positive droplets (N_+_) were counted. The concentration of bacteria (C) was determined as C = λ/ν, where λ is the average number of bacteria per droplet, which can be calculated by −ln (1 − N_+_/N), and ν is the average volume of the droplets. For comparison of digital droplet-FISH count with the reference count, the expected bacterial concentration (reference count) from the initial culture was determined using disposable hemocytometer chips (C-Chips DHC-N01, NanoEnTek, Seoul, Korea) by following the manual instruction (cell/mL = average count per square × dilution factor × 10^4^ (volume factor)) under the microscope (Axio Observer, ZEISS, Oberkochen, Germany) using 400× magnification.

## 3. Results and Discussion

### 3.1. Microfluidic One-Pot Digital Droplet-FISH Assay

Our microfluidic one-pot (e.g., wash-free) digital droplet-FISH assay is an amplification-free assay performed in picoliter-droplets using CP1 to permeabilize bacterial cell membranes and permit LNA/DNA MBs to hybridize with 16S rRNA, which generates a fluorescence signal allowing bacteria detection (Figure 1B). This assay circumvents all washing steps that typically exists in conventional FISH assays and can absolutely quantify bacteria by digital counting.

To design the microfluidic one-pot and wash-free digital droplet-FISH assay, a high fluorescence signal-to-noise (S/N) ratio is of importance to differentiate the fluorescence signal from MB-binding bacteria and the background within the droplet, which still contain MBs with quenched fluorescence signals. In addition, the one-pot droplet-FISH assay is a direct probe-target binding assay without amplification of the target sequences, so the fluorescence signal is limited to the gene abundance of the bacteria. Since our target is bacterial 16S rRNA and the cellular abundance of rRNA varies between bacterial species (e.g., *Mycobacterium tuberculosis* have 10^2^–10^3^ copies of rRNA per bacterial cell, whereas *E. coli* may have as many as 10^4^–10^5^ copies [44]), those species with a low copy number of rRNA might lead to insufficient signal intensities for detection. Therefore, a higher fluorescence intensity is also preferable for signal detection and direct visualization of bacteria in droplets. For the above reasons, optimization of the hybridization of the MBs is essential.

Detailed optimization process for hybridization conditions is reported in the Supplementary Materials. We found that LNA/DNA MB with 3 stems, ATTO647N as the fluorophore, and BHQ-2 as the quencher hybridized with synthetic templates in a urea-NaCl-based hybridization buffer displayedthe highest S/N ratio and fluorescence intensity (Figure S4). Furthermore, to distinguish single mismatch sequences and provide better specificity, a hybridization temperature of 70 °C was chosen (Figure S5). After the determination of the hybridization temperature, the concentration of LNA/DNA MBs have to be adapted for the low template concentration prevailing in a single bacterium. The results showed that 25 nM of LNA/DNA MBs hybridized with 10 nM synthetic template or a bacterial crude lysate provided the highest S/N ratio for the tested range of concentrations (Figure S6). Therefore, 25 nM of 3 stem LNA/DNA MBs containing a ATTO647N fluorophore and a BHQ-2 quencher hybridized with bacteria in a urea-NaCl-based hybridization buffer at 70 °C was used in our one-pot droplet-FISH assay.

### 3.2. Microfluidic One-Pot Digital Droplet-FISH Assay for Bacteria Detection at the Single Cell Level

**Figure 2:**
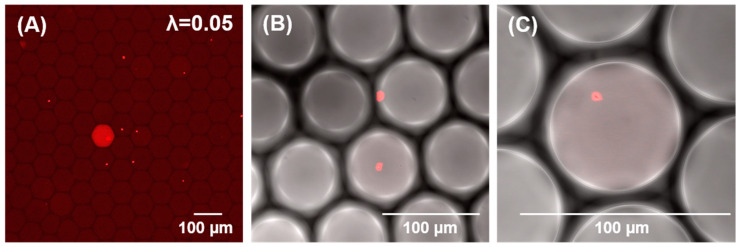
Microfluidic one-pot digital droplet-FISH assay for bacteria detection at the single cell level. Single bacterium encapsulation in droplets. The red fluorescent dot represents a single *E. coli*. (**A**) magnification: ×100, (**B**) magnification: ×400, (**C**) magnification: ×630.

After the optimization of the LNA/DNA MBs and hybridization conditions, the assay was integrated into a microfluidic one-pot digital droplet-FISH assay for bacterial detection. *E. coli* were encapsulated in 210 pL droplets where *in situ* permeabilization and *in situ* hybridization took place. To ensure single cell encapsulation (either 0 or 1 bacteria per droplet), the expected mean occupancy (λ) should be ≤0.1 [45,46]. In this experiment, the mean occupancy of bacteria per droplet was 0.05. This means that approximately 5% of total droplets yielded a single bacterium. As shown in Figure 2, singularization of the bacteria could be achieved. Furthermore, higher magnification of the microscopic images revealed clearly that the fluorescence signal was emitted from a single bacterium inside a droplet (Figure 2B,C) and that positive droplets (yielding a single *E. coli*) could be differentiated from negative droplets (no bacterial cells inside the droplets), which makes this technique suitable for a digital enumeration assay. In the following section, the results of digital quantification are presented. 

The applied permeabilizer, CP1, is a linear cationic α-helical peptide that disrupts the membrane by orienting parallel to the surface of the lipid bilayer and forming pores (carpet mechanism) [47]. CP1 was shown to be highly effective in causing permeabilization of the membrane of bacteria [48]. In the case of the Gram-negative bacteria demonstrated here, CP1 permeabilizes both the outer membrane (primarily a coat of lipopolysaccharide) and the inner membrane (comprised of phospholipid) [49] so that LNA/DNA MBs can diffuse into the bacteria and bind to bacterial 16S rRNA (Figure 1B). It has been demonstrated that CP1-treated *E. coli* had pores ranging from 4.2–9.6 nm [50]. We then calculated the hydrodynamic radius (R_h_) of the presented LNA/DNA MB byliterature [51] and found the size of the LNA/DNA MBs (R_h_ = 0.04 nm) is much smaller than the pores. Furthermore, there is a peptidoglycan layer between the outer and inner membranes of Gram-negative bacteria that is accessible to molecules with the molecular weight in the range 30–57 kDa [52]. Since the molecular weight of our LNA/DNA MB is 7.27 kDa, the LNA/DNA MBs should easily diffuse and pass through the porous peptidoglycan layer. Accordingly, we observed bacteria presenting a fluorescence signal throughout the entire bacterial cell (Figure 2), which proved the accessibility of LNA/DNA MB for bacterial cells.

As shown in Figure 2A, some droplets displayed a fluorescence signal within the entire droplet, and in some of the droplets, only bacteria had the fluorescence signal. The presence of a fluorescence signal in the whole droplet likely implies that some free bacterial 16S rRNA was released through the pore of the bacterial cell membrane. The formation and dissociation of pores of certain size are random [53]. Therefore, when the pores in the outer and inner membrane are sufficiently large, it may mediate the outflow of cytoplasmic contents [50] into the droplet, resulting in the red signal observed in the whole volume of the droplet. In FISH assays, it is necessary to ensure sufficient cell permeability for the probes to enter and bind to the target site. However, cell permeabilization needs to be carefully balanced in order to maintain cell integrity [54]. It has been shown that the disruption of bacterial cell membranes by CP1 is dose-dependent [55]. Although adding more CP1 could possibly increase the outflow of 16S rRNA and fluorescence signal distribution inside the droplets, the concentration of CP1 should not exceed a critical concentration. If the concentration of the permeabilizer is too high, it could lead to complete lysis of the bacteria or earlier perforation of the bacterial cell wall, which would result in a reduced bacterial concentration by false compartmentation, and therefore, false enumeration. To adapt a simple fluorescence detection system to the one-pot droplet-FISH assay instead of confocal microscopy, other permeabilizers with the ability to form increased pore size diameters while maintaining cell integrity could be implemented into the assay. By decreasing the droplet size in combination with optimal permeabilizers and stronger fluorophores, the local fluorescence intensity per droplet/cavity could be increased due to the concentration effect [45], and hence, the signal intensity would be stronger for a simple instrument readout.

### 3.3. Absolute Quantification of Bacteria by Microfluidic One-Pot Digital Droplet-FISH Assay

**Figure 3:**
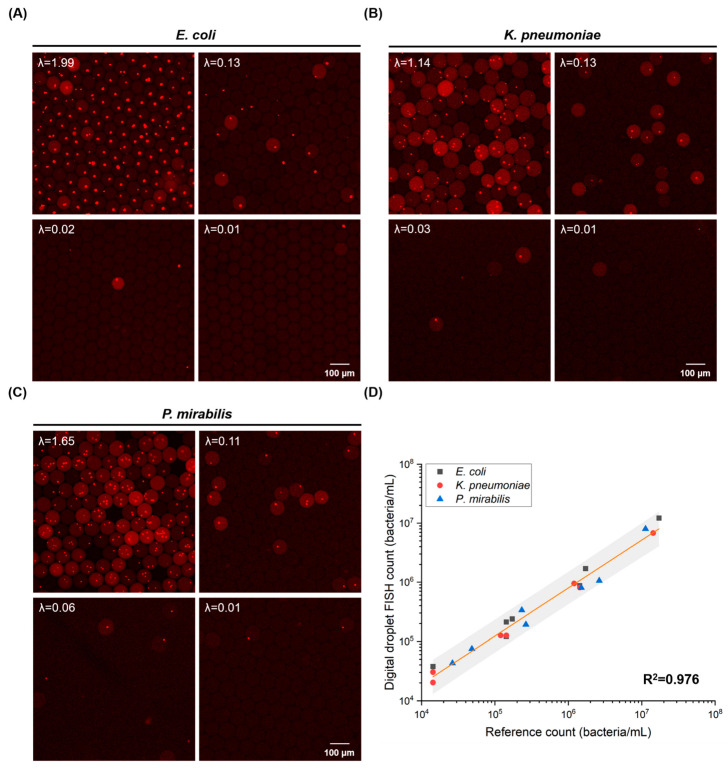
Absolute quantification of bacteria by droplet-FISH assay. (**A**) Representative fluorescence images of different concentrations of *E. coli*. The average number of *E. coli* per droplet is presented as λ. (**B**) Representative fluorescence images of different concentrations of *K. pneumoniae*. The average number of *K. pneumoniae* per droplet is presented as λ. (**C**) Representative fluorescence images of different concentrations of *P. mirabilis*. The average number of *P. mirabilis* per droplet is presented as λ. (**D**) Correlation plot for bacterial concentration measured by one-pot droplet-FISH against the expected bacterial concentration (reference count) for the different bacterial species. Every point represents one bacterial concentration. The orange line is the linear fit based on all data points, hence, it includes variation for all three tested bacterial species. The slope of the fitting line is 0.81 ± 0.03, and the intercept is 1.03 ± 0.16. The gray band is the 95% confidence interval.

We applied three bacteria species inculding *E. coli*, *K. pneumonia*, and *P. mirabilis* to demonstrate absolute quantification of bacteria in our one-pot droplet-FISH assay. We calculated the ratio of positive droplets to the total droplets in fluorescence images (Figure 3A–C) to determine the value of λ, and used the Poisson formula described in the Materials and Methods to obtain the bacterial concentration (referred as the digital droplet-FISH count). Our results showed a good correlation between the digital droplet-FISH counts and the expected bacterial concentration counted by hemocytometer for bacterial loads ranging from 1.43 × 10^4^ bacteria/mL to 1.72 × 10^7^ bacteria/mL. The coefficient of determination (R^2^) for all data points including variation in the three tested bacteria species was 0.976 (R^2^ = 0.976) (Figure 3D). Individually, the R^2^ for *E. coli* was 0.998, for *K. pneumonia* was 0.997, and for *P. mirabilis* was 0.987. The results showed the compatibility and repeatability of the microfluidic one-pot digital droplet-FISH assay for different strains of bacteria. 

Here, we directly compared the true quantity (also known as the population value in statistics) [56] from the reference count with the digital droplet-FISH count to avoid propagation errors from serial dilutions, especially at low concentrations. In fact, even lower concentrations could be detected by the microfluidic one-pot digital droplet-FISH assay (data not shown). However, due to the inherent limitation of a hemocytometer, it was difficult to count bacterial concentrations lower than 10^4^ bacteria/mL accurately for comparison with digital droplet-FISH counts. The chamber of a hemocytometer has a total volume of 0.1 mm^3^, which gives a volume factor of 10^4^. The number of bacteria per square is multiplied by this volume factor. Therefore, when the bacteria concentration is lower than 10^4^ bacteria/mL, the accuracy of a hemocytometer is very limited. Aside from a microscopic count using a hemocytometer, a standard plate count (SPC) for colony forming units might not be suitable for reference count in this study. A SPC only counts viable bacteria growing as a CFU, but a digital droplet-FISH count enumerates the total bacterial load, which includes both live and dead (or non-active/ non-dividing) bacterial cells.

In our experimental setup, the LabDisk generated about 47,600 droplets with a volume of ~210 pL, which resulted in a lower limit of quantification (LLOQ) of 3 × 10^2^ bacteria/mL and an upper limit of quantification (ULOQ) of 4.6 × 10^7^ bacteria/mL in theory. This calculation is based on the assumption that a sample would have a 95% chance of generating at least one positive droplet at the LLOQ and a 95% chance of generating at least one negative droplet at the ULOQ [57] (refer to Supplementary Materials: formula (2)–(6)). Therefore, the ideal dynamic range of quantification is five orders of magnitude. However, due to the current LabDisk design, we limited our evaluation to the area where droplets were present and arranged in a monolayer [41]. Therefore, we only analyzed 4000 to 5000 droplets in each reaction chamber in one large image scan, so the theoretical LLOQ was set at around 3 × 10^3^ bacteria/mL and the ULOQ was at around 3 × 10^7^ bacteria/mL, resulting in a proven dynamic range of quantification with only four orders of magnitude. Further optimization of LabDisk regarding the pyramidal structure [58] in the reaction chamber is needed in order to analyze all droplets. By analyzing more droplets, the dynamic range can be expanded because the dynamic range of quantification is proportional to the number of analyzed droplets in the digital assays. In addition to analyzing more droplets in one reaction, the dynamic range could also be expanded by integrating the one-pot droplet-FISH assay with a dual-volume centrifugal step emulsification cartridge [59], which produces small-volume droplets to enable the quantification of high concentrations, and large-volume droplets enable the quantification at low concentrations. Furthermore, adopting optimized digital quantification statistics [26,60] is another way to increase the ULOQ. In the case of samples containing a low number of bacteria (such as a water sample), a filtration/concentration step could be incorporated to expand the LLOQ with high sensitivity. In fact, the sensitivity of quantification in a relevant dynamic range for the target bacteria in the samples is essential. To meet the needs of different applications, customized microfluidic cartridges for specific applications that generate theon-demand size and number of droplets should be considered.

The presented one-pot FISH assay (wash-free *in situ* permeabilization and hybridization in one-step) could work not only with the LabDisk but also other droplet microfluidic systems for the digital enumeration of bacteria. This assay could also work without encapsulation of bacteria in droplets. For example, to quantify the bacteria in water where filtration is essential to concentrate the sample, our one-pot FISH assay could possibly be incorporated with a hydrophobic grid membrane filter [61] or asymmetric membrane [62] that allows random bacterial distribution and isolation into grid compartments through filtration. As a result, digital quantification of the bacteria with Poisson statistics could also be applied.

## 4. Conclusions

In summary, we have developed a microfluidic one-pot digital droplet-FISH assay to simplify the conventional FISH protocol [63] (Figure S7) with reduced manual handling steps and time-to-results for absolute quantification of bacteria. The users only need to mix the bacteria, MBs, and permeabilizers in the hybridization buffer, and then load into an automated centrifugal microfluidic system for cell encapsulation, *in situ* permeabilization and *in situ* hybridization. This assay is straightforward and can be finished in 1.5 hours (including quantification). The time for quantification could be shortened if we could speed up image analysis with artificial neural networks using multiple training algorithms to count the cells [64]. In addition, we optimized the working conditions ofLNA/DNA MBs to improve the S/N ratio and used CP1 to allow the access of LNA/DNA MBs to bacterial 16S rRNA inside the bacteria. These strategies enabled bacteria detection at the single cell level without any amplification steps for the target templates. Furthermore, we quantified *E. coli*, *K. pneumonia*, and *P. mirabilis* as a demonstration and showed a correlation of R^2^ = 0.976 between the digital droplet-FISH count and the bacterial count by a hemocytometer.

The presented microfluidic one-pot digital droplet-FISH assay showed its potential for quantifying the total amount of bacteria in samples with the possibility of enumerating VBNC bacteria. Although we only designed universal MBs for bacterial detection and enumeration in this study, we assume that our method is directly transferable to selective enumeration and identification of bacteria in mixed cultures. By designing LNA/DNA MBs for targeting species-specific variable regions in the 16S rRNA of bacteria and incorporating these LNA/DNA MBs with different fluorophores/quencher combinations, multiplex FISH analysis should be possible with our microfluidic one-pot digital droplet FISH method.

## Data Availability

Not applicable.

## Supplementary Materials

The following supporting information can be downloaded at: https://www.mdpi.com/article/10.3390/bios12040237/s1, Figure S1: The microfluidic setup for partitioning of the sample [41]; Figure S2: Example image showing the droplet recognition and size analysis using customized MATLAB program [42]; Figure S3: The demonstration of counting positive droplets by Fiji Cell Counter; Figure S4: Screening of the designed LNA/DNA MBs in different hybridization buffers [65,66,67,68,69]; Figure S5: Optimization of the hybridization temperature for better specificity [70]; Figure S6: Determination of the concentration of LNA/DNA MB [44,71,72]; Figure S7: Comparison of the FISH protocols from Cold Spring Harbor protocols^12^ with one-pot droplet-FISH [63]; Table S1: The sequence of LNA/DNA MBs targeting domain *Bacteria*; Table S2: The sequences of the synthetic templates (lower case: mismatch bases) [73]; Table S3: Different composition of hybridization buffer [37,57,74].
