# Supplementary material for: Microfluidic One-Pot Digital Droplet FISH Using LNA/DNA Molecular Beacons for Bacteria Detection and Absolute Quantification
Source: Biosensors (Basel). 2022 Apr 12;12(4):237. doi: 10.3390/bios12040237 (PMC9032532; doi:10.3390/bios12040237)
Supplement: Supplementary file 1 [file biosensors-12-00237-s001.zip › biosensors-1646947-supplementary.pdf]

Electronic supplementary information (ESI):
Microfluidic digital droplet-FISH using LNA/DNA molecular beacons enabling one-pot detection and absolute quantification of bacteria

Yu-Ting Kao, [a, b] Silvia Calabrese, [c] Nadine Borst, [a, c] Michael Lehnert, [c] Yu-Kai Lai, [a, b, c] Franziska Schlenker, [c] Peter Juelg, [c] Roland Zengerle, [a, c] Piotr Garstecki, [b] and Felix von Stetten [a, c, *]

[a] Laboratory for MEMS Applications, IMTEK - Department of Microsystems Engineering, University of Freiburg, Georges-Koehler-Allee 103, 79110 Freiburg, Germany

[b] Institute of Physical Chemistry, Polish Academy of Sciences, Kasprzaka 44/52, 01-224 Warsaw, Poland.

[c] Hahn-Schickard, Georges-Koehler-Allee 103, 79110 Freiburg, Germany

Supplemental figures

1. Microfluidic setup.

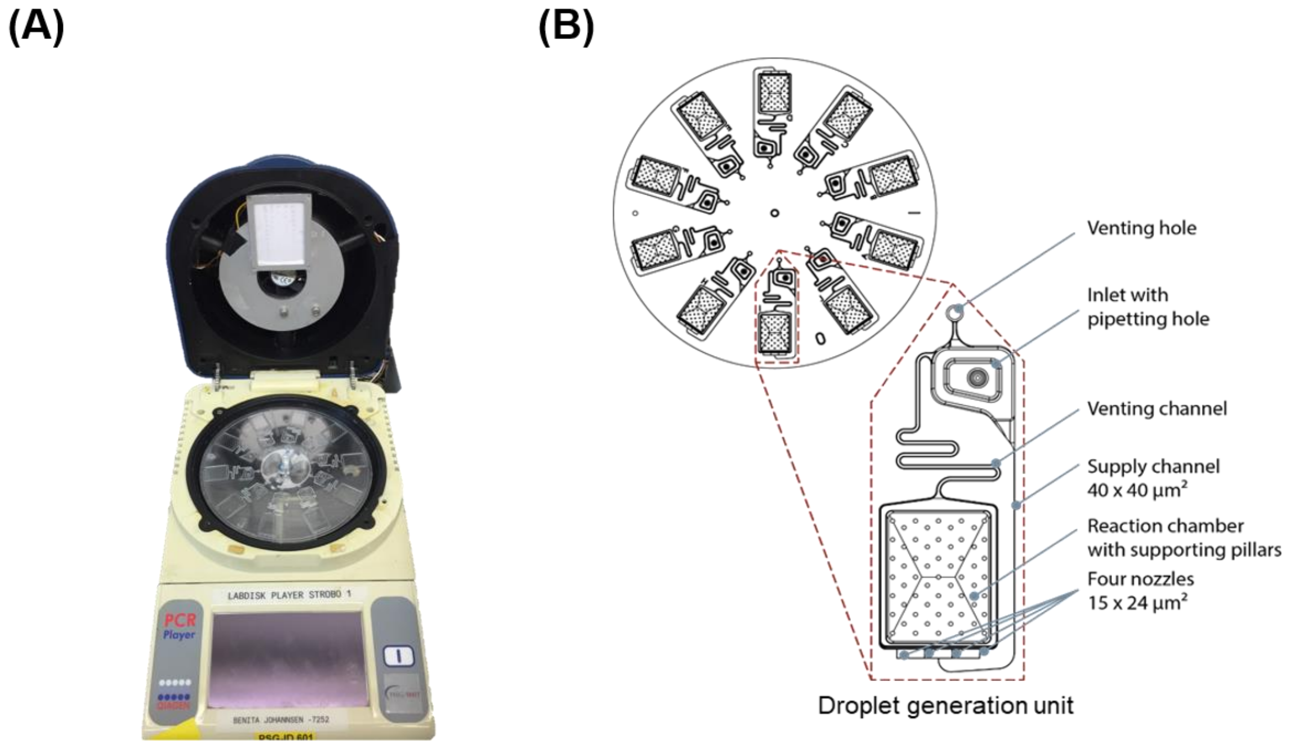

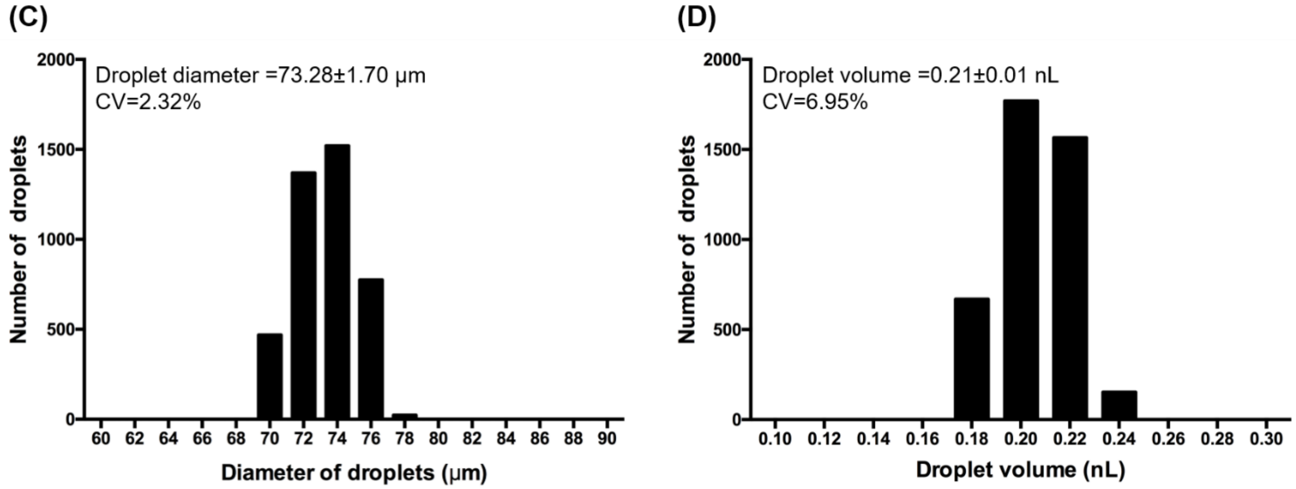

Figure S1 The microfluidic setup for partitioning of the sample. (A) The image of LabDisk Player 1. A customized centrifuge equipped with temperature control system. **(B) CAD Layout of LabDisk and description of fluidic components[1].** The LabDisk features 10 droplet generation units and each unit is capable to process an individual assay. It is manufactured in foil technology by the Hahn-Schickard foundry service. The structured part of the LabDisk is made from a 300 µm cyclic olefin copolymer foil (COC 8007/COC 6013; Tekniplex, USA) and it is thermally sealed by a 200 µm COC foil. To ensure the chamber depth of 40 µm in the center and 80 µm at the rim structure, supporting pillars are included in the reaction chamber to avoid the touchdown of the sealing foil to the structured part. The venting holes on the backside of the disk are closed with an adhesive PTFE membrane (Porex, USA) whereas the pipetting holes in the inlet are sealed with a pressure sensitive adhesive foil (9795R, 3M, USA) after pipetting. A supply channel (width and depth 40 µm) connects the inlet with the reaction chamber. Four nozzles with a width of 24 µm and a depth of 15 µm are used for droplet generation. **(C) Size distribution of the produced droplets in LabDisk.** The diameter of the droplet is 73.28±1.70 µm. **(D) Volume distribution of the produced droplets in LabDisk.** The volume of the droplet is 0.21±0.01 nL.

2. Droplet recognition and size analysis using customized MATLAB program

Customized MATLAB script[2] was used to detect and analyze the size of the droplets. Bright field images were first converted to grey image, and followed by build-in circle detection function. As shown in the example image (**Figure S2**), the detected droplets are marked with red circle. The diameter of the droplets was calculated by pixel/µm ratio given by the microscope. Note: the big circles are the supporting pillar structures in the reaction chamber.

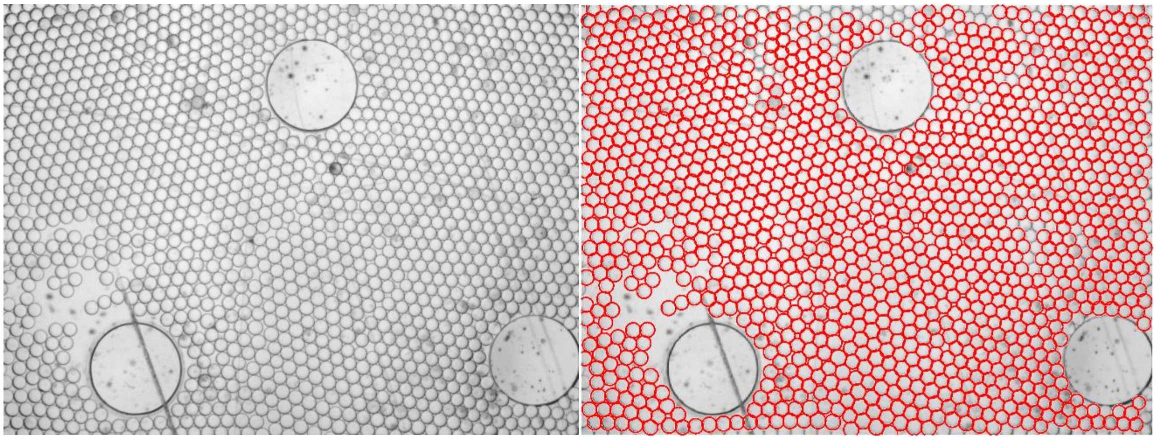

Figure S2 Example image showing the droplet recognition and size analysis using customized MATLAB program.

3. Counting positive droplets by Fiji

Demonstration of counting positive droplets by Fiji is shown in **Figure S3**. Type 1is defined as 1 bacteria in droplet, Type 2 is 2 bacteria in droplet, Type 3 is 3 bacteria in droplet, etc. We counted the total number of droplets that contains bacteria no matter how many bacteria inside and referred it as positive droplet.

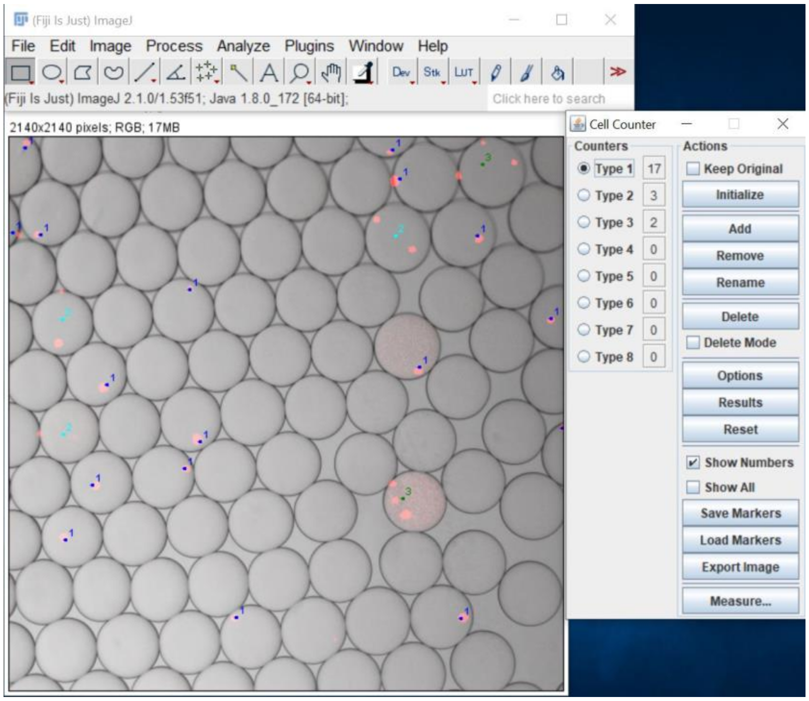

Figure S3 The demonstration of counting positive droplets by Fiji Cell Counter.

4. Optimization of hybridization conditions for one-pot droplet FISH assay

4.1 Screening of LNA/DNA MBs and hybridization buffer

To find out the optimal composition for droplet-FISH assay, different LNA/DNA MBs were designed (**Table S1**) and analyzed in different hybridization buffers (**Table S3**), which were used for LNA-FISH assay in the literatures. Here, synthetic templates complementary to MBs (**Table S2**) were used as target sequences in this experiment. In order to achieve good binding properties with high S/N ratios, six MBs (P2556-1, P2556-2, P2655, P2656, P2600, P2601) targeting the 16S rRNA in the domain *Bacteria* designed with different stem lengths, incorporation of LNA bases for improved binding properties, as well as varying fluorophore/quencher combinations were evaluated.

To differentiate the real signal generated by MB-target binding from the background signal, it is suggested that the MBs with the S/N ratio (formula (1)) above 20 is preferable when 5-fold molar excess of synthetic templates were adding into the reaction[3]. In such condition, we observed P2655, P2656, and P2600 in HBF-1 and P2655, P2656, P2600 and P2601 in HBF-3 exhibited the S/N ratio above 20 (**Figure S4**). We further compared the fluorescence intensity and found P2600 had the highest fluorescence intensity in HBF-3. Therefore, LNA/DNA MB (P2600) with 3 stems, ATTO647N as fluorophore and BHQ-2 as quencher in HBF-3 was used for the following experiments.

Several factors influence the S/N ratio for the MBs, which render proper MB design critical. MBs with too long or too short stems both resulted in low S/N ratio[4]. In addition, it has been shown that the different fluorophore and quencher combination of the hairpin molecules would affect the S/N ratio and fluorescence signal[5]. Comparing P2556-2 and P2600 which have the same LNA/DNA sequences but with different fluorophores and quenchers, we found that P2600 performed higher S/N ratio and fluorescence intensity (**Figure S4**) in three hybridization buffers. This result is in line with the findings in real-time PCR using universal reporter oligonucleotides[5]. Furthermore, when comparing P2655 with P2656, P2655 consisting of FAM as fluorophore and BHQ-1 as quencher displayed higher S/N ratio than P2656 which used FAM / BMN-Q-535 combination in all buffer conditions. Moreover, we observed that the composition of the hybridization buffer also plays a role in influencing the S/N ratio and fluorescence intensity. Urea-based hybridization buffer has been shown to have improved S/N ratio[6] and signal intensity[7] comparing with the formamide containing buffer in *in situ* hybridization method. Corresponding to literature

findings, our results showed that LNA/DNA MBs had overall higher S/N ratios and fluorescence intensity in urea-based hybridization buffer (HBF-3) than the others which are Tris-NaCl based or formamide containing buffer (**Table S3**).

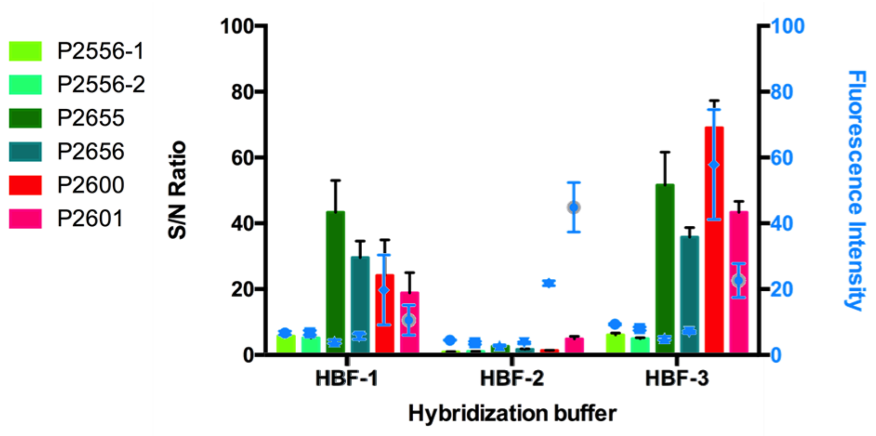

Figure S4 Screening of the designed LNA/DNA MBs in different hybridization buffers. The fluorescence intensity of 6 different 100 nM LNA/DNA MBs in different hybridization buffers (HBF-1, HBF-2, and HBF-3) in the presence (500 nM) of their corresponding target sequence measured at 55°C after 30 minutes hybridization on a thermal cycler. Experiments were performed by three repetitions and repeated on three independent days. The error bar is the propagation error.

4.2 Determination of hybridization temperature

To increase the assay specificity, an optimal hybridization temperature is necessary. In order to achieve this, the hybridization temperature of P2600 to the target template was determined by the fluorescence derivative curves (**Figure S5B**), which were derived from thermal denaturation profiles (**Figure S5A**). Furthermore, those fluorescence derivative curves allow the detection of single point mutations by melting temperature shifts and can give information about specificity of the designed MB[8]. By analyzing the fluorescence derivative curves, we identified the melting temperature of P2600 hybridized with the perfect match was 72.5°C while with mismatch-1 was 52.5°C and with mismatch-2 was 65.8°C (**Figure S5B**). In order to distinguish the single mismatch sequences to have better specificity, 70°C was chosen to be the hybridization temperature.

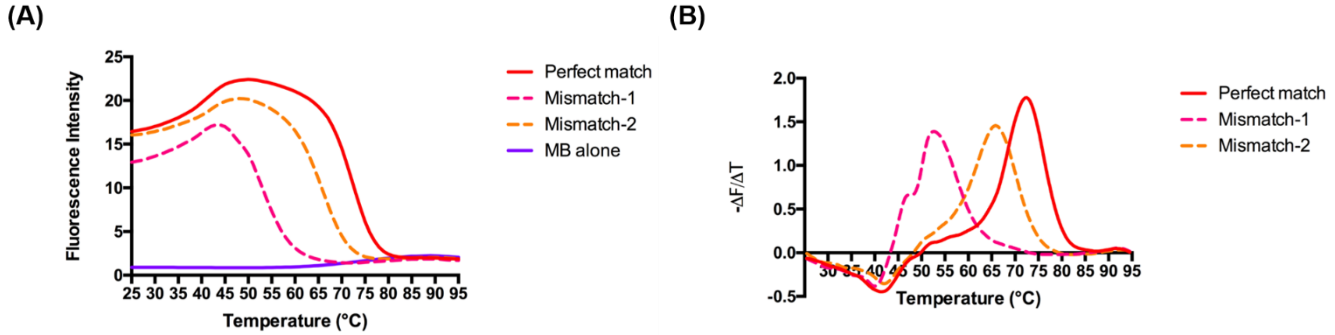

Figure S5 Optimization of the hybridization temperature for better specificity. (A) The thermal denaturation profile of P2600 in HBF-3. **(B)** The fluorescence derivative curves of P2600 in HBF-3. 100 nM P2600 hybridize with 500 nM perfect match sequences or mismatches in HBF-3. The presented data were the average of three repetitions.

4.3 Optimizing the concentration of LNA/DNA MB for detection of low concentration of templates

Compared to 500 nM of synthetic templates that we used for previous experiments, the concentration of the target templates is much less than in real bacteria sample. Here, we used 10 nM synthetic templates ($\approx 1.2\times10^{11}$ copies in 20 µl reaction mix) to mimic the 16S

rRNA copy numbers of 10^8 CFU/mL *E. coli* assuming each *E. coli* contain 10^4–10^5 copies of 16S rRNA[9]. A range of P2600 concentration from 25 nM to 500 nM were tested with 10 nM synthetic templates in HBF-3 for the evaluation of S/N ratios (**Figure S6A).** The result showed that 25 nM P2600 hybridized with 10 nM synthetic templates had 2-fold higher S/N ratio compared to the others.

Due to the variation of 16S rRNA gene copy numbers in various bacteria species[10,11], we examined 6 different bacteria species such as *S. aureus*, *S. epidermidis*, *S. agalactiae*, *E. coli*, *K. pneumoniae*, and *P. mirabilis* to assess the effect of lowering the concentration of LNA/DNA MBs on S/N ratio. Using crude bacteria lysate as the templates, we observed that 25 nM P2600 exhibited much higher S/N than 200 nM P2600 when reacting with different crude bacteria lysates (**Figure S6B**). Therefore, 25 nM P2600 was chosen for the one-pot droplet-FISH assay, in which it was aimed to target single bacterium in those microcavities. These results also showed the universal bacteria targeting characteristic of the designed LNA/DNA MBs.

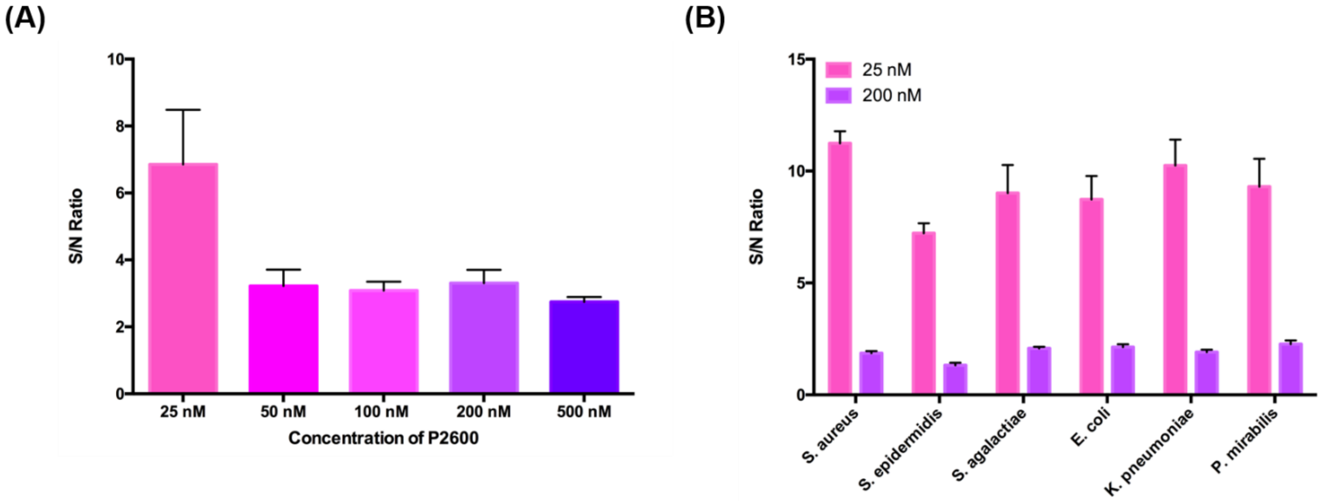

Figure S6 Determination of the concentration of LNA/DNA MB. (A) The S/N of different concentration of P2600 hybridize to 10 nM synthetic template. **(B)** Comparison of the S/N ratio of 25 nM and 200 nM MB in ~10^8 CFU/mL crude bacteria lysate. Error bars represent standard deviation of three replicates.

5. Comparison of the FISH protocols from Cold Spring Harbor protocols with one-pot droplet-FISH.

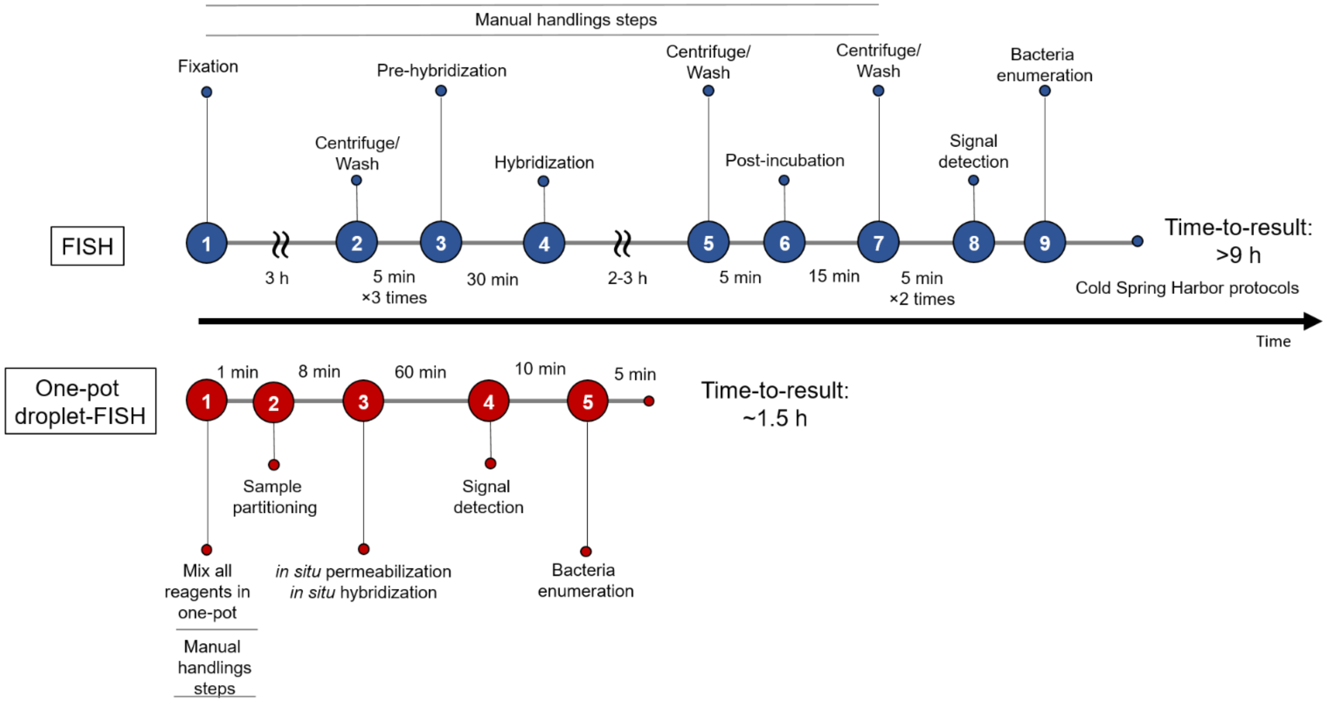

Figure S7 Comparison of the FISH protocols from Cold Spring Harbor protocols[12] with one-pot droplet-FISH. One-pot droplet-FISH assay simplifies the lengthy and labour-intensive conventional FISH protocol. The time-to-result of one-pot droplet-FISH for the detection and enumeration of bacteria is 1.5 hours (5000 droplets were analyzed for absolute bacterial quantification).

Supplemental tables and formula

1. The design of LNA/DNA MBs

Table S1 The sequence of LNA/DNA MBs targeting domain *Bacteria*. Nucleotides in bold target the domain S-D-Bact-0338-a-A-18; letters in red are LNA monomers; letters in black are DNA monomers; and underline nucleotides form a stem structure due to self-complementary.

LNA/DNA MBs	Sequences (5'-3')
P2556-1	FAM-TCC**GCTGCCTCCCGTAGGA**-DABCYL
P2556-2	FAM-TCC**GCTGCCTCCCGTAGGA**-DABCYL
P2655	FAM-ACTC**GCTGCCTCCCGTAGGAGT**-BHQ-1
P2656	FAM-ACTC**GCTGCCTCCCGTAGGAGT**-BMN-Q-535
P2600	ATTO647N-TCC**GCTGCCTCCCGTAGGA**-BHQ-2
P2601	ATTO647N-ACTC**GCTGCCTCCCGTAGGAGT**-BHQ-2

2. Evaluation of signal-to-noise ratios of LNA/DNA MBs in different hybridization buffer

The signal-to-noise (S/N) ratio was determined by the following formula:

$$S/N = \frac{F_{open} - F_{buffer}}{F_{close} - F_{buffer}} \tag{1}$$

where, the rise in fluorescence was monitored until it reached a stable level as F_{open}. The fluorescence intensity of the MBs without perfect match is F_{close} and F_{buffer} is the fluorescence intensity of the hybridization buffer only[3,13]. Experiments were run in a thermal cycler (Rotor-Gene Q, QIAGEN, Germany) where the signals were recorded at 55°C for 30 minutes. 100 nM LNA/DNA MBs and 500 nM complementary sequence (**Table S2**) were hybridized in different hybridization buffers listed in **Table S3**.

3. The sequences of the synthetic templates

Synthetic templates listed in **Table S2** were either designed to mimic perfectly the LNA/DNA MB binding side (perfect match) or to test selectivity of designed MB by introducing single nucleotide exchanges (mismatch). The synthetic templets were synthesized by biomers.net (Ulm, Germany).

Table S2. The sequences of the synthetic templates (lower case: mismatch bases)

Templates	Sequences (5'-3')
Perfect match	ACTCCTACGGGAGGCAGC
Mismatch-1	ACTCCTACGcGAGGCAGC
Mismatch-2	ACTCgTACGGGAGGCAGC

4. The hybridization buffers

Three composition of hybridization buffers that used in LNA-FISH assay in the literature are listed in **Table S3**.

Table S3. Different composition of hybridization buffer

Hybridization buffer	Composition	Reference
HBF-1	20 mM Tris-HCl (pH 7.5)	Yang *et al.*[14]

	5 mM $MgCl_2$<br>50 mM NaCl	
HBF-2	2X saline-sodium citrate (SSC)<br>10% Dextran (pH 7.0)<br>50% Formamide	Silahtaroglu *et al.*[15]
HBF-3	0.02 mM Tris-HCl (pH 7.0)<br>900 mM NaCl<br>1 M urea	Lawson *et al.*[7]

5. Lower limit of quantification (LLOQ) and upper limit of quantification (ULOQ)[16]

Poisson distribution:

$p(k) = \frac{Cv^k \cdot e^{-Cv}}{k!}$, and for k=0 (empty droplets), $p = e^{-Cv}$ (2)

The Poisson distribution gives the probability, *p*, that there are *k* bacteria in a given droplet based on an average concentration per droplet, *Cv*, where *v* is the volume (mL) of droplet and C is the bulk bacteria concentration (CFU/mL).

The number of negative droplet, N_-, out of the total droplets, N, can serve as the estimation of *p*. Therefore, the observed results can be used to calculate the concentrations.

$$C = -\frac{\ln(\frac{N_-}{N})}{v} \quad (3)$$

The binomial equation is used to determine the probability, *P*, that a specific experimental result will be observed.

$$P = \binom{N}{N_-} \cdot p^{N_-} \cdot (1-p)^{N-N_-}$$

$$= \binom{N}{N_-} \cdot (e^{-Cv})^{N_-} \cdot (1-e^{-Cv})^{N-N_-} \quad (4)$$

where $\binom{N}{N_-} = \frac{N!}{N_-!(N-N_-)!}$

The LLOQ is defined as the concentration that would have a 95% chance of generating at least one positive compartment.

Assume no positive droplets in the chamber: $N - N_- = 0 \rightarrow N = N_-$

$$P = \binom{N}{N} \cdot (e^{-Cv})^N \cdot 1 = e^{-CvN}$$

If the probability of having at least one positive droplet is 95%, then

$$0.95 = 1 - e^{-CvN}$$

$$C = \frac{-\ln(0.05)}{vN} \quad (5)$$

Therefore, the LLOQ can be presented as formula (5).

The ULOQ is defined as the concentration, which would have a 95% chance of generating at least one negative compartment.

Assume no negative droplets in the chamber: $N_- = 0$

$P = \binom{N}{0} \cdot 1 \cdot (1 - e^{-Cv})^N = (1 - e^{-Cv})^N$

If the probability of having at least one negative droplet is 95%, then

$0.95 = 1 - (1 - e^{-Cv})^N$

$$C = \frac{-\ln(1 - \sqrt[N]{0.05})}{v} \qquad (6)$$

Therefore, the ULOQ can be presented as formula (6)

References

(1) Schlenker, F.; Kipf, E.; Borst, N.; Paust, N.; Zengerle, R.; Stetten, F. von; Juelg, P.; Hutzenlaub, T. Centrifugal Microfluidic Integration of 4-Plex ddPCR Demonstrated by the Quantification of Cancer-Associated Point Mutations. *Processes* **2021**, *9* (1), 97. DOI: 10.3390/pr9010097.

(2) Schuler, F.; Siber, C.; Hin, S.; Wadle, S.; Paust, N.; Zengerle, R.; Stetten, F. von. Digital droplet LAMP as a microfluidic app on standard laboratory devices. *Anal. Methods* **2016**, *8* (13), 2750–2755. DOI: 10.1039/C6AY00600K.

(3) Zhu, Z. Design, Synthesis, Purification, and Characterization of Molecular Beacons. In *Molecular beacons*; Yang, C. J., Ed.; Springer, 2013; pp 1–17. DOI: 10.1007/978-3-642-39109-5_1.

(4) Tsourkas, A.; Behlke, M. A.; Rose, S. D.; Bao, G. Hybridization kinetics and thermodynamics of molecular beacons. *Nucleic acids research* **2003**, *31* (4), 1319–1330. DOI: 10.1093/nar/gkg212.

(5) Lehnert, M.; Kipf, E.; Schlenker, F.; Borst, N.; Zengerle, R.; Stetten, F. von. Fluorescence signal-to-noise optimisation for real-time PCR using universal reporter oligonucleotides. *Anal. Methods* **2018**, *10* (28), 3444–3454. DOI: 10.1039/c8ay00812d.

(6) Sinigaglia, C.; Thiel, D.; Hejnol, A.; Houliston, E.; Leclère, L. A safer, urea-based in situ hybridization method improves detection of gene expression in diverse animal species. *Developmental biology* **2018**, *434* (1), 15–23. DOI: 10.1016/j.ydbio.2017.11.015. Published Online: Dec. 9, 2017.

(7) Lawson, T. S.; Connally, R. E.; Vemulpad, S.; Piper, J. A. Dimethyl formamide-free, urea-NaCl fluorescence in situ hybridization assay for Staphylococcus aureus. *Letters in applied microbiology* **2012**, *54* (3), 263–266. DOI: 10.1111/j.1472-765X.2011.03197.x. Published Online: Jun. 1, 2012.

(8) Huang, Q.; Liu, Z.; Liao, Y.; Chen, X.; Zhang, Y.; Li, Q. Multiplex fluorescence melting curve analysis for mutation detection with dual-labeled, self-quenched probes. *PloS one* **2011**, *6* (4), e19206. DOI: 10.1371/journal.pone.0019206. Published Online: Apr. 28, 2011.

(9) Stender, H.; Fiandaca, M.; Hyldig-Nielsen, J. J.; Coull, J. PNA for rapid microbiology. *Journal of Microbiological Methods* **2002**, *48* (1), 1–17. DOI: 10.1016/S0167-7012(01)00340-2.

(10) Klappenbach, J. A.; Saxman, P. R.; Cole, J. R.; Schmidt, T. M. rrndb: the Ribosomal RNA Operon Copy Number Database. *Nucleic acids research* **2001**, *29* (1), 181–184. DOI: 10.1093/nar/29.1.181.

(11) Větrovský, T.; Baldrian, P. The variability of the 16S rRNA gene in bacterial genomes and its consequences for bacterial community analyses. *PloS one* **2013**, *8* (2), e57923. DOI: 10.1371/journal.pone.0057923. Published Online: Feb. 27, 2013.

(12) Parsley, L. C.; Newman, M. M.; Liles, M. R. Fluorescence in situ hybridization of bacterial cell suspensions. *Cold Spring Harbor protocols* **2010**, *2010* (9), pdb.prot5493. DOI: 10.1101/pdb.prot5493. Published Online: Sep. 1, 2010.

(13) Zhang, B.; Maimaiti, Y.; Liu, C.; Li, J.; Wang, H.; Lin, H.; Deng, Z.; Lu, X.; Zhang, X. Direct detection of Staphylococcus aureus in positive blood cultures through molecular beacon-based fluorescence in situ hybridization. *Journal of Microbiological Methods* **2019**, *159*, 34–41. DOI: 10.1016/j.mimet.2019.02.007.

(14) Yang, C. J.; Wang, L.; Wu, Y.; Kim, Y.; Medley, C. D.; Lin, H.; Tan, W. Synthesis and investigation of deoxyribonucleic acid/locked nucleic acid chimeric molecular beacons. *Nucleic acids research* **2007**, *35* (12), 4030–4041. DOI: 10.1093/nar/gkm358.
(15) Silahtaroglu, A. N.; Tommerup, N.; Vissing, H. FISHing with locked nucleic acids (LNA): evaluation of different LNA/DNA mixmers. *Molecular and Cellular Probes* **2003**, *17* (4), 165–169. DOI: 10.1016/S0890-8508(03)00048-3.
(16) Kreutz, J. E.; Munson, T.; Huynh, T.; Shen, F.; Du, W.; Ismagilov, R. F. Theoretical design and analysis of multivolume digital assays with wide dynamic range validated experimentally with microfluidic digital PCR. *Analytical chemistry* **2011**, *83* (21), 8158–8168. DOI: 10.1021/ac201658s. Published Online: Oct. 7, 2011.
